# Multimodal Feedback-Enhanced Soft Hand Rehabilitation Robot: System Development and Preliminary Clinical Evaluation

**DOI:** 10.3390/bioengineering13101172

**Published:** 2026-10-08

**Authors:** Jianwei Lai, Aiguo Song, Xuemei Fan, Guangyu Sun, Yaxuan Di, Ting Wu

**Affiliations:** 1 The State Key Laboratory of Digital Medical Engineering, Jiangsu Key Lab of Remote Measurement and Control, School of Instrument Science and Engineering, Southeast University, Nanjing 210096, China; 101300484@seu.edu.cn; 2 East China Institute of Photo-Electronic Integrated Device Suzhou Branch Center, Suzhou 232101, China; merry_fxm@163.com (X.F.); sun_sgy@163.com (G.S.); 3 Department of Neurology, The First Affiliated Hospital of Nanjing Medical University, Nanjing 210029, China; diax@nju.edu.cn (Y.D.); wuting80000@126.com (T.W.)

**Keywords:** soft rehabilitation robotics, lattice-structured soft pneumatic actuator, multimodal feedback, human–robot interaction, hand rehabilitation, stroke rehabilitation

## Abstract

Soft robotic systems have attracted increasing attention for hand rehabilitation because of their compliance, adaptability, and potential for comfortable human–robot interaction. However, the clinical translation of soft hand rehabilitation robots remains limited by insufficient integration of interactive training, user feedback, and practical wearability. This study presents a multimodal feedback-enhanced soft hand rehabilitation robot based on lattice-structured soft pneumatic actuators (LSPAs), together with a preliminary clinical evaluation. The system integrates a fabric-based soft glove, pneumatic actuation, fingertip force sensing, hand-motion tracking, and interactive virtual rehabilitation tasks to provide coordinated visual, auditory, force-related, and mirror-visual feedback. Material characterization was first conducted to compare three TPU materials with different mechanical properties, followed by system-level evaluation of usability, wearability, and actuator durability. A four-week robot-assisted rehabilitation intervention was then conducted in nine stroke survivors. Participants completed five one-hour sessions per week, including passive training and multimodal interactive training. Clinical outcomes were evaluated using the Brunnstrom motor recovery stage, Fugl–Meyer Assessment of the upper extremity (FMA-UE), National Institutes of Health Stroke Scale (NIHSS), and Activities of Daily Living (ADL) scale, while usability was assessed using the System Usability Scale (SUS). After four weeks, the mean Brunnstrom stage increased from 2.44±2.07 to 3.67±2.12, FMA-UE increased from 18.33±25.63 to 32.67±23.11, NIHSS decreased from 5.67±4.36 to 3.22±2.99, and ADL increased from 49.78±39.53 to 66.67±22.50. The mean SUS score was 74.4. TPU420D provided the best durability among the tested configurations, with more than 30 h of recorded use without failure during the experimental period. These findings support the feasibility and usability of the proposed system and provide preliminary evidence for its application in stroke hand rehabilitation. Because of the small sample size, heterogeneous post-stroke status, short intervention period, and absence of a control group, the clinical findings should be interpreted as preliminary rather than confirmatory evidence of therapeutic efficacy.

## 1. Introduction

Stroke is a leading cause of hand motor dysfunction among the elderly population [1], significantly impairing activities of daily living and overall quality of life. Many stroke survivors require long-term assistance from caregivers, thereby imposing substantial physical, emotional, and economic burdens. Conventional rehabilitation therapies are often constrained by limited training intensity, insufficient dosage, and high costs, which restrict their effectiveness and accessibility. These limitations have driven increasing interest in the development of robotic-assisted rehabilitation systems [2,3,4].

Recent advances in rehabilitation robotics have incorporated diverse technologies, including remote rehabilitation platforms [5], passive spring-based training systems [6], hand force-feedback gloves [7], functional electrical stimulation (FES) [8], and virtual reality (VR)-based interaction [9]. Remote rehabilitation enables higher training intensity beyond clinical settings and can achieve outcomes comparable to conventional face-to-face therapy [10]. However, passive devices, force-feedback gloves, and VR-based systems often rely on residual motor function, making them less suitable for patients with severe impairments, particularly in the absence of therapist supervision [11,12,13].

The integration of robotic assistance with interactive technologies such as VR has enabled the delivery of high-intensity and high-dose rehabilitation in home-based environments [14]. For instance, Nijenhuis et al. developed a remote rehabilitation system based on passive spring and elastic tension mechanisms [15], allowing patients to engage in therapist-guided training remotely after initial setup [16]. These systems have demonstrated improvements in clinical outcomes, including the Fugl–Meyer Assessment (FMA), Box and Block Test (BBT), Action Research Arm Test (ARAT), and Jebsen–Taylor Hand Function Test (JTHFT). Nevertheless, most existing studies primarily rely on clinical evaluation metrics and provide limited investigation into user-centered factors such as patient engagement, usability, and acceptance, which are critical for long-term rehabilitation adherence.

Although some rigid hand exoskeletons have reached commercialization and undergone preliminary clinical validation [17], they often suffer from limited wearability, poor adaptability to varying hand anatomies, and high manufacturing costs [18], thereby constraining their application in home-based rehabilitation scenarios. In contrast, soft robotic systems offer inherent compliance, improved safety, and enhanced user comfort [19]. In particular, textile-based soft robots have attracted increasing attention due to their low stiffness, high adaptability, and potential for improved human–machine interaction [20,21,22]. Despite these advantages, current soft hand rehabilitation systems remain insufficiently validated in real-world environments, especially with respect to usability, patient acceptance, and sustained engagement outside controlled laboratory settings [23].

Alternative compliant structures include an FDM-printable tendon-driven continuum robot with a serial S-shaped backbone [24] and a fishbone-inspired continuum robot with rigid–flexible–soft coupling [25]. These studies illustrate different approaches to compliant bending. Recent work also integrated flexible sensors with a fine-tuned multimodal model for shape recognition and fault diagnosis in a six-bar tensegrity structure [26]. Its sensing and state-estimation task differs from patient feedback during rehabilitation, but provides context for future state monitoring.

Building upon our previous work on high-performance hand rehabilitation robots [27,28], this study proposes a soft hand rehabilitation robot system incorporating a multimodal feedback-driven human–machine interaction framework. The system integrates a fabric-based soft glove with interactive rehabilitation scenarios that combine visual, auditory, and task-oriented feedback to enhance sensorimotor engagement during training. The design and system architecture of the robot and its training platform are presented, followed by evaluations of usability, wearability, and preliminary therapeutic outcomes. The evaluation provides preliminary evidence that the proposed system is feasible and well-accepted by stroke participants, while supporting further investigation of its clinical utility.

## 2. Materials and Methods

### 2.1. Material Characterization and Selection for the LSPAs

**Figure 1:**
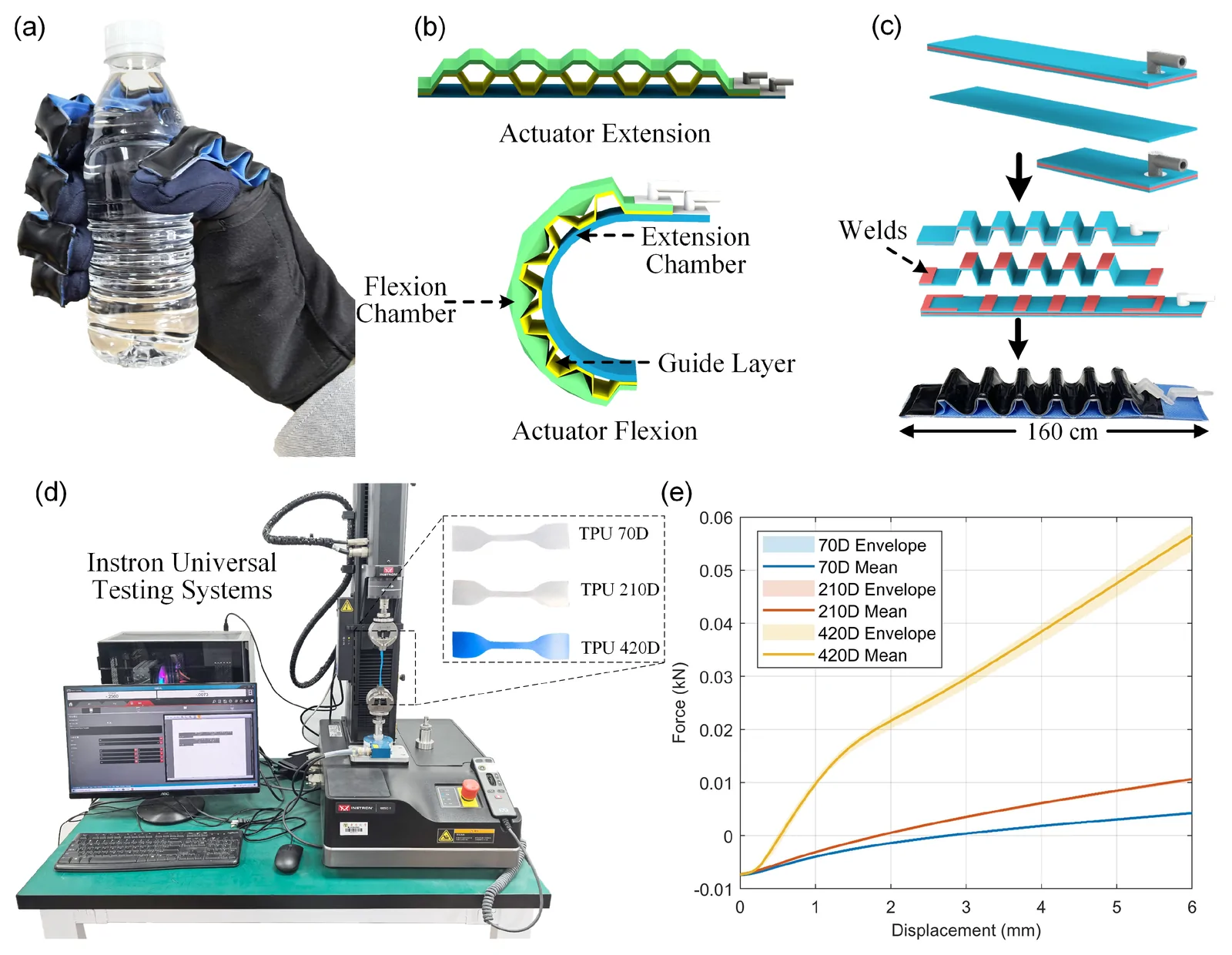
Material design and characterization of a lattice-structured soft hand rehabilitation robot. (**a**) Soft hand rehabilitation robot integrating LSPAs. (**b**) LSPA structure and bidirectional actuation states. (**c**) Fabrication process of the LSPA. (**d**) Uniaxial tensile testing setup for three TPU materials. (**e**) Force–strain curves of TPU70D, TPU210D, and TPU420D, showing mean and envelope curves.

The materials used in the fabrication of lattice-structure soft pneumatic actuators (LSPAs) play a critical role in determining the performance, stability, and durability of soft rehabilitation robots. To enhance the adaptability and actuation efficiency of the proposed system, the constituent materials of the LSPAs were carefully selected and systematically evaluated. As illustrated in Figure 1a, the proposed soft hand rehabilitation robot consists of an open-design cotton glove integrated with five LSPAs. The open glove configuration facilitates donning and doffing for patients with finger movement disorders, thereby improving the overall usability and adaptability of the rehabilitation system.

The LSPAs are fabricated using a thermoplastic polyurethane (TPU) composite fabric material, which comprises a fabric substrate and a TPU functional layer. The fabric substrate is woven in a ring structure, providing a relatively high elastic modulus while maintaining low bending stiffness. This structural characteristic contributes to improved mechanical stability and shape retention of the actuator during pressurization. In contrast, the TPU functional layer exhibits a smooth surface and excellent thermoplastic properties, enabling reliable sealing through thermal bonding. The synergistic combination of the fabric substrate and TPU layer effectively prevents air leakage during pneumatic inflation and ensures stable, repeatable actuation performance.

The structure of the LSPA is illustrated in Figure 1b. The actuator consists of a flexion chamber, a guiding layer, and an extension chamber. These three layers operate cooperatively to achieve bidirectional assisted finger motion. The flexion chamber, fabricated from TPU materials, serves as the primary driving component for finger flexion. Upon pneumatic inflation, the flexion chamber expands and generates a flexion torque, thereby assisting the patient in performing finger flexion movements. The guiding layer is fabricated from a single-layer TPU material and serves to reduce relative friction between the flexion and extension cavities while constraining excessive deformation of both layers. This constraint ensures accurate actuation direction and enhances motion stability. The extension chamber, which is also fabricated from double-layer TPU materials, generates an extension driving force upon inflation, assisting the finger in completing extension movements. By alternately inflating and deflating the flexion and extension chambers, bidirectional assisted finger motion is achieved, thereby satisfying the fundamental requirements of finger rehabilitation training.

The LSPA is fabricated using a hot-pressing process based on TPU materials, and the manufacturing procedure is illustrated in Figure 1c. First, the extension chamber and guiding layer are pre-bent to a predefined curvature to ensure conformity with the natural motion trajectory of the finger. Subsequently, the flexion chamber, guiding layer, and extension chamber are precisely aligned and simultaneously thermally bonded at predetermined welding locations to form a compact multilayer structure. This fabrication method simplifies the assembly process while enhancing structural integrity and coordination among the actuation layers.

The LSPA retains the structural design and dimensions of the honeycomb pneumatic actuator (HPA) developed in our previous work [28]. That study reported an actuator tip force of 13.57 N at 150 kPa. During its glove-assisted patient evaluation, MCP and PIP joint angles were $87.67\pm 19.27^{\circ }$ and $64.20\pm 30.66^{\circ }$, respectively, and mean fingertip force was 10.16±4.24 N. These values are previously reported results, not new measurements from the present cohort. To enhance the wearing comfort and long-term durability of the LSPA in soft hand rehabilitation robots, uniaxial tensile tests were conducted on the actuator materials to guide material selection. Three TPU materials with different Shore hardness levels—TPU70D, TPU210D, and TPU420D—were evaluated. The experiments were performed using an INSTRON universal testing machine, as shown in Figure 1d. Each test was repeated three times to reduce experimental uncertainty.

The results of the uniaxial tensile tests are summarized in Figure 1e, which presents the displacement–force curves. The figure shows the mean curve obtained from three trials, together with the envelope of the experimental results. The Young’s moduli of TPU70D, TPU210D, and TPU420D were measured as 62.81 MPa, 92.47 MPa, and 375.36 MPa, respectively, indicating substantial differences in mechanical properties. TPU70D exhibited a maximum load of 19.51 N at a tensile strain of 41.13%, while TPU210D reached a peak load of 30.23 N at a strain of 40.00%. In contrast, TPU420D achieved a significantly higher maximum load of 59.4 N at a comparatively lower strain of 24.73%. These results demonstrate that TPU materials with higher Shore hardness possess greater load-bearing capacity under tensile loading. Conversely, TPU materials with lower Shore hardness are more sensitive to pneumatic pressure and undergo larger deformations at lower pressures, which is advantageous for realizing compliant actuation.

Based on the uniaxial tensile test results and the functional requirements of different actuator components, a differentiated material selection strategy was adopted. The extension chamber must provide sufficient actuation force to ensure that the fingers reach the desired extension angle; therefore, TPU420D, with its higher stiffness and load-bearing capacity, was selected for the extension chamber. In contrast, the flexion chamber must balance durability, wearing comfort, and force output compatibility. To address these multidimensional requirements, LSPAs with flexion cavities fabricated from TPU420D, TPU210D, and TPU70D were evaluated through comparative experiments. This systematic evaluation enables optimization of the flexion actuator design, thereby better satisfying the practical requirements of finger flexion rehabilitation training.

### 2.2. Design of the Rehabilitation System Based on Multimodal Feedback

**Figure 2:**
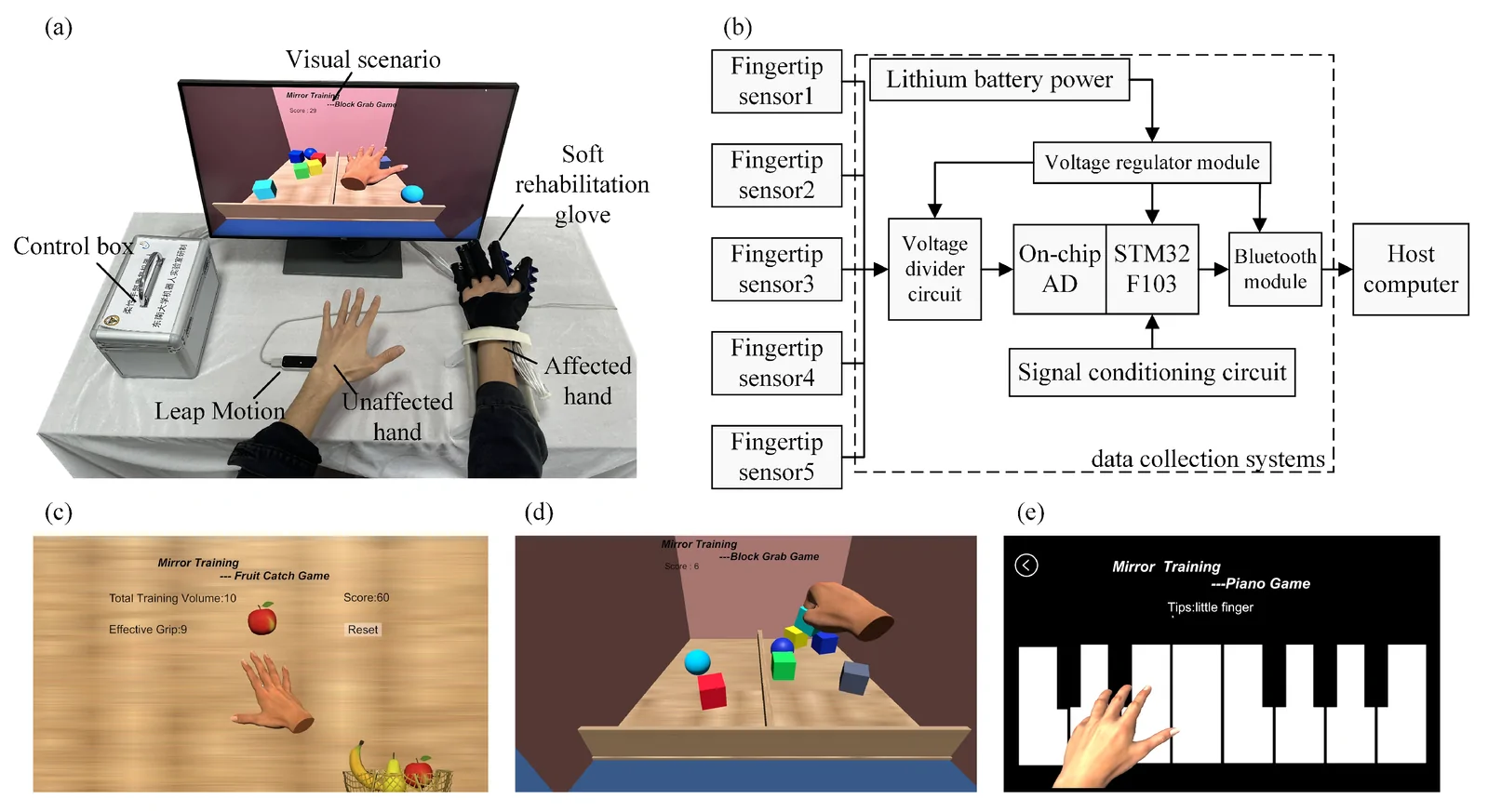
Design of the multimodal feedback–based soft hand rehabilitation robot system. (**a**) System composition including robot, control box, glove, virtual scene, and sensors. (**b**) Control block diagram of the robot control box. (**c**) Virtual interactive scenario: grasping fruit, training vertical spatial awareness. (**d**) Virtual interactive scenario: grasping wooden blocks, training horizontal spatial awareness. (**e**) Virtual interactive scenario: playing piano, training fine motor skills.

This study developed a soft hand rehabilitation robot system based on multimodal feedback, as illustrated in Figure 2a. The system comprises a robot control box, a soft rehabilitation glove, a virtual environment running on a computer, Leap Motion sensors, and an arm support frame. The soft glove is connected to the control box via pneumatic lines, enabling communication with the internal controller for actuator control. The Leap Motion sensor is used to capture finger interaction motions, while the virtual environment displays the real-time interaction between the patient and the virtual scene.

The system block diagram of the pneumatic control box is shown in Figure 2b. The main controller of the control box is an STM32F405 microcontroller, which acquires fingertip force sensor signals through an analog-to-digital (AD) module. The control circuit integrates a pulse-width modulation (PWM) control module for driving the air pump and solenoid valves, as well as circuits for air pressure sensing. In addition, the control system incorporates fingertip force signal conditioning, voltage monitoring and alarm functions, reverse diode protection, electrostatic discharge protection, reverse polarity protection, and communication and switching interfaces between the control box and the computer. The maximum commanded operating pressure was set to 150 kPa in the control program. A software stop button was provided to allow the operator to stop system operation when needed. The performance of these functions under fault conditions was not quantitatively evaluated in this study.

The multimodal feedback framework designed in this study is shown in Figure 2c–e. The system provides users with multiple forms of feedback, including force, tactile, visual, auditory, and mirror visual illusion feedback. It is primarily intended for hemiplegic patients with unilateral limb impairment, who are required to perform interactive movements with the unaffected hand during rehabilitation training. During therapy, the patient’s healthy fingers interact with a virtual environment, while the Leap Motion sensor captures finger kinematics and transmits this information simultaneously to a host computer and a slave computer. The host computer runs rehabilitation games within a virtual environment, in which the captured finger motions control virtual finger movements to complete task-specific interactions and receive performance-based scores. The interactive training scenarios include three representative tasks: fruit grasping, wooden block manipulation, and piano playing. These tasks are designed to train vertical spatial perception, horizontal spatial perception, and fine motor control of individual fingers, respectively. Meanwhile, the slave computer generates control commands for the soft hand rehabilitation robot to assist synchronous movements of the patient’s affected fingers. Throughout the rehabilitation process, the system provides real-time visual and auditory feedback through the rehabilitation games. To promote active participation, task completion is accompanied by score-based feedback and encouraging voice prompts, such as “Great job!” and “Congratulations on passing the level!”.

## 3. Clinical Evaluation

### 3.1. Clinical Protocol

**Figure 3:**
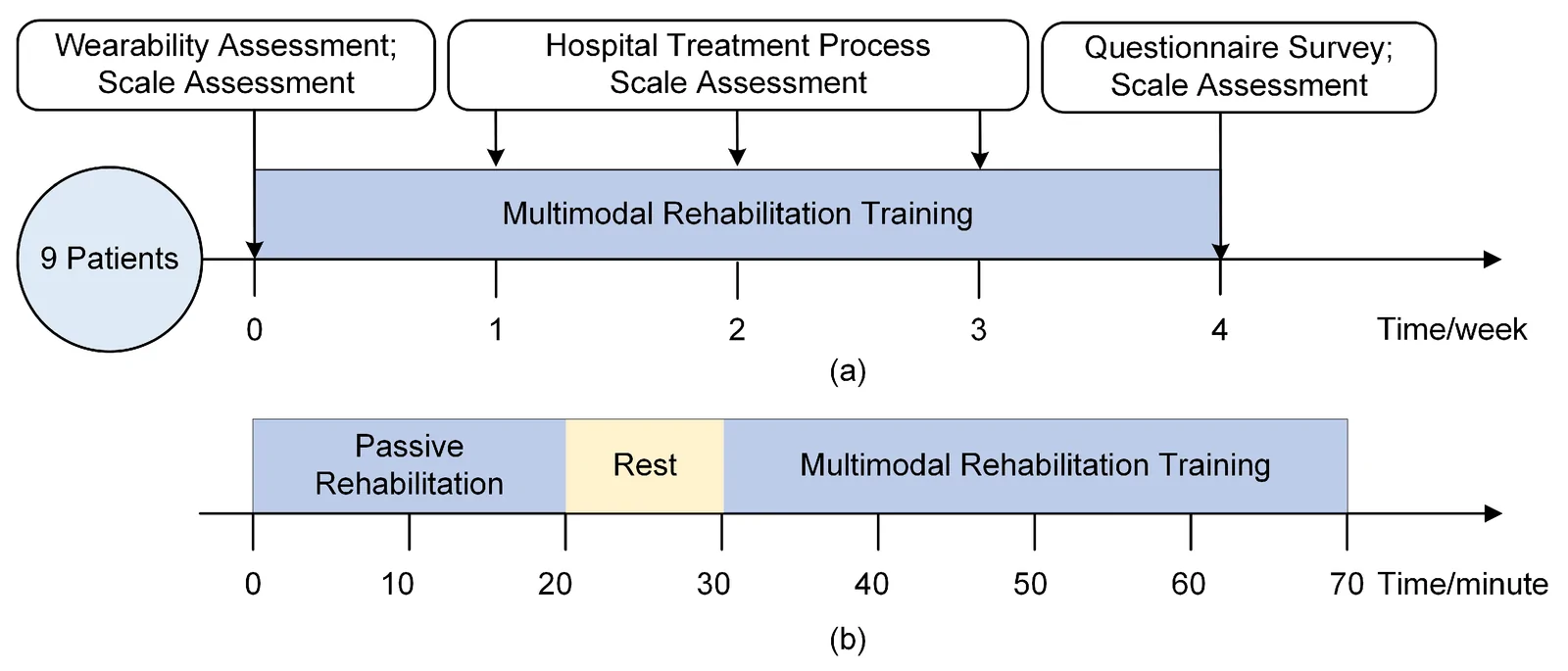
Intervention workflow for the multimodal feedback-based soft hand rehabilitation robot. (**a**) Overall clinical intervention flowchart. (**b**) Specific treatment components in a single session.

The clinical trial was conducted at Jiangsu Provincial People’s Hospital. Participants were enrolled in a 4-week intervention program, and the overall study protocol is illustrated in Figure 3. Baseline assessments were performed at the hospital prior to the intervention and included standardized clinical scales and robotic measurements of hand grip strength. All baseline evaluations were administered by licensed therapists.

Throughout the trial, all participants received rehabilitation therapy under the supervision of a physician and did not undergo any conventional rehabilitation interventions during the study period. Before the commencement of training, therapists evaluated each participant’s finger motor function and recorded the corresponding clinical scale scores as baseline data. Participants then completed four consecutive weeks of robot-assisted rehabilitation therapy, with clinical assessments conducted at the end of each week.

Rehabilitation sessions were conducted five times per week, with a scheduled duration of 70 min per session, including rest. Two therapists were present to supervise operation and provide guidance. Participants were encouraged to don the glove independently, with caregiver assistance when needed.

Each session consisted of three phases. During the first 20 min, participants underwent passive rehabilitation training, in which the soft hand rehabilitation robot assisted the affected fingers in performing repetitive grasping movements to promote finger mobility and reduce muscle tension. This was followed by a 10 min rest period to mitigate muscle fatigue caused by continuous motion. Subsequently, participants engaged in 40 min of multimodal rehabilitation training using the soft hand rehabilitation robot system. The specified phases total 70 min per session: 60 min of active training and 10 min of rest.

Upon completion of the 4-week intervention, all participants completed a questionnaire survey to evaluate the usability of the multimodal-feedback-based soft hand rehabilitation robot system. In parallel, therapists collected qualitative feedback by soliciting participants’ subjective experiences and suggestions during the rehabilitation process, which were used to further improve system performance and clinical applicability.

### 3.2. Inclusion Criteria for Participants

The inclusion criteria for this study were as follows: (i) age between 18 and 75 years; (ii) ability to correctly understand and follow the therapist’s instructions; and (iii) a confirmed diagnosis of stroke within three years before enrollment. Participants were excluded if they met any of the following conditions: (i) severe cognitive impairment, defined as a Mini-Mental State Examination (MMSE) score below 23; (ii) presence of open wounds on the affected limb; (iii) pain experienced during assessment of the affected limb; (iv) excessive finger muscle tone, defined as a Modified Ashworth Scale (MAS) score greater than 3; or (v) receipt of additional upper-limb rehabilitation interventions during the study period.

### 3.3. Clinical Test Indicators

Standardized clinical assessment scales were employed to evaluate the effects of robot-assisted rehabilitation on patients’ finger motor function. These included the Brunnstrom motor recovery stage, the Fugl–Meyer Assessment for the upper extremity (FMA-UE), the National Institutes of Health Stroke Scale (NIHSS), and the Activities of Daily Living (ADL) scale.

To assess patients’ subjective experience and motivation during rehabilitation training, questionnaire-based evaluations were conducted using Likert-scale instruments. System usability was assessed using the System Usability Scale (SUS) [29]. SUS scores range from 0 to 100, with higher scores indicating better perceived usability; a score above 68 is generally considered indicative of acceptable usability. Each questionnaire item was rated on a 5-point Likert scale, ranging from 1 (“strongly disagree”) to 5 (“strongly agree”).

To evaluate the wearing comfort and tolerance of the soft hand rehabilitation gloves, three glove variants were fabricated. All gloves employed lattice-structured actuators, with the extension and guiding layers fabricated from TPU420D, while the flexion layer was fabricated from TPU70D, TPU210D, or TPU420D. The gloves were randomly assigned to participants, and the wearing duration and total usage time associated with each material configuration were recorded and statistically analyzed.

Due to the relatively small sample size, statistical analysis was performed using the Wilcoxon matched-pairs signed-rank test to assess differences in clinical and robotic measurements. A significance level of p<0.05 (denoted by *) was adopted. Because the present manuscript reports the descriptive clinical changes rather than a complete set of inferential statistics, the clinical findings are interpreted primarily as preliminary trends. All data analyses were conducted using MATLAB R2022a.

## 4. Clinical Trial

### 4.1. Participant Characteristics

**Figure 4:**
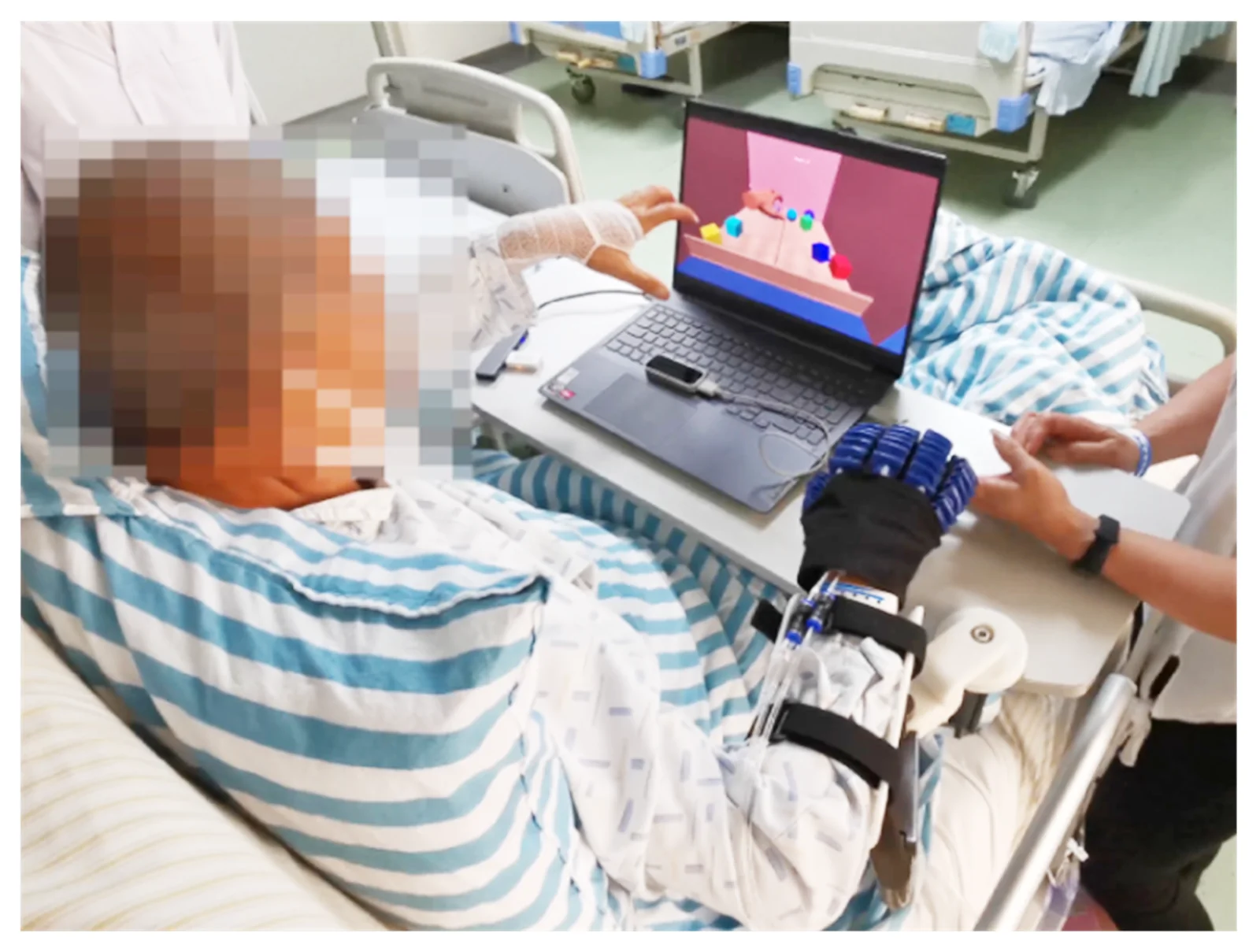
Clinical treatment setup showing a patient using the multimodal feedback-based soft hand rehabilitation robot system.

All participants who met the inclusion criteria were enrolled in this clinical trial, and their demographic and clinical characteristics are summarized in Table 1. A total of nine stroke survivors were recruited, including 4 women and 5 men, with a mean age of 58.1±11.75 years. Participants were recruited from the Department of Neurology at Jiangsu Provincial People’s Hospital. All participants provided written informed consent prior to participation. The study protocol was approved by the Ethics Review Committee of Jiangsu Provincial People’s Hospital (Approval No. 2020-SR-362). The experimental training scenario is illustrated in Figure 4.

### 4.2. Clinical Outcome Assessment

**Figure 5:**
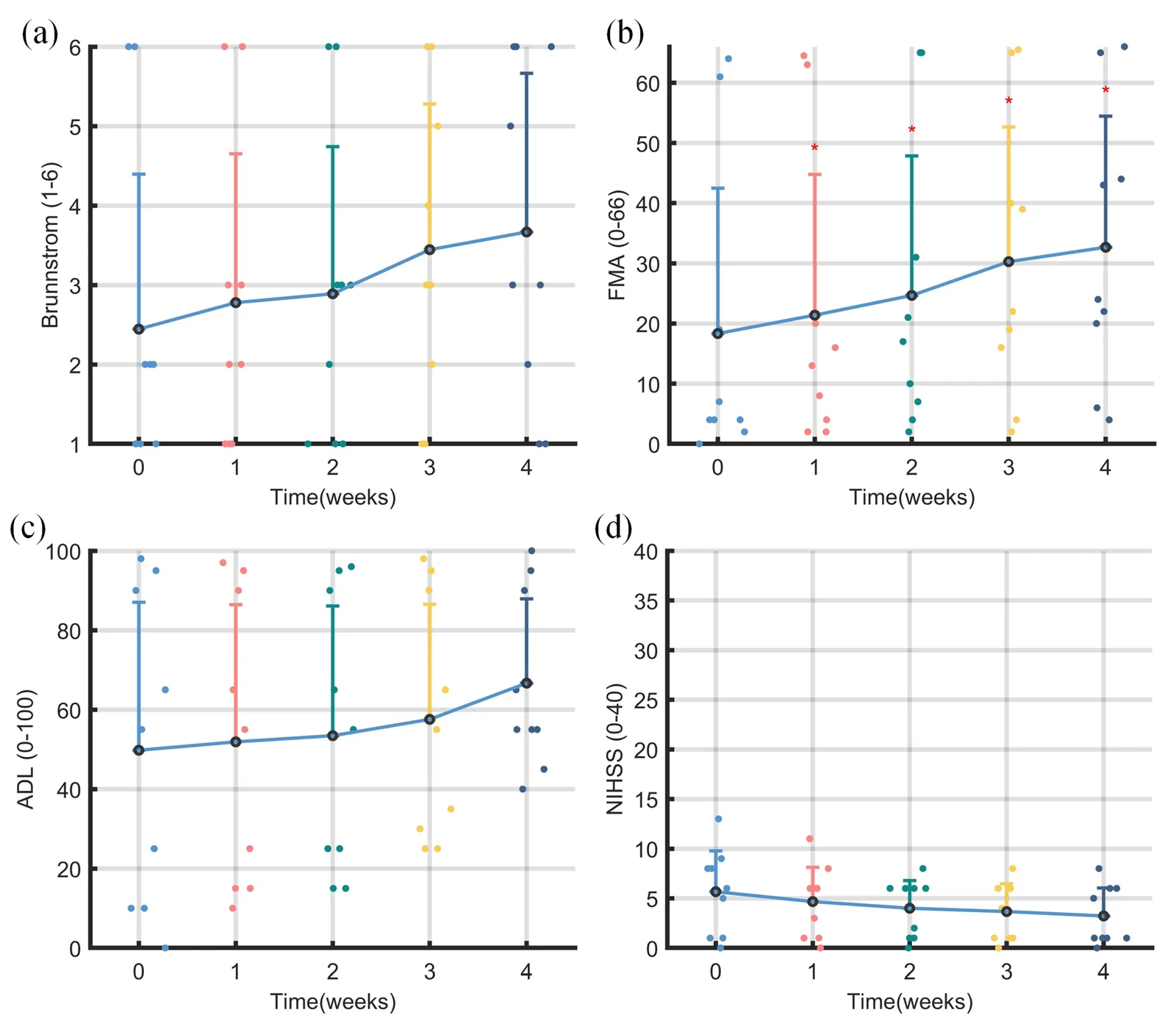
Clinical assessment results of participants across four scales: (**a**) Brunnstrom, (**b**) FMA, (**c**) ADL, and (**d**) NIHSS. Error bars represent standard deviation.

All participants completed the planned rehabilitation protocol in the study period. No participant withdrew from the study due to discomfort or difficulties associated with wearing the soft rehabilitation glove. Clinical scale data were collected and analyzed, and the corresponding results are presented in Figure 5.

Changes in Brunnstrom motor recovery stage are shown in Figure 5a. At baseline, Patients 1 and 2 were at Stage VI, while the remaining seven participants were at Stage I or II. The median stage (interquartile range) was II (I–II) at baseline, II (I–III) at week 1, III (I–III) at week 2, III (II–V) at week 3, and III (II–VI) at week 4. From baseline to week 4, four participants advanced to a higher stage, five remained at the same stage, and none moved to a lower stage. Patient 9 showed the largest observed stage change, from Stage II to Stage VI. These descriptive changes cannot be attributed to the intervention in the absence of a control group.

Changes in the Fugl–Meyer Assessment (FMA) scores are illustrated in Figure 5b. At baseline, Patients 1 and 2 exhibited relatively high FMA scores of 61 and 64, respectively, whereas the remaining participants demonstrated more severe motor impairment. Following four weeks of robot-assisted rehabilitation, upper limb motor function improved across the cohort, with mean FMA scores increasing from 18.33±25.63 to 32.67±23.11. In particular, Patient 4 showed an increase in FMA score from 4 to 43, and Patient 9 improved from 4 to 44. Improvements in FMA scores were observed as early as the first week of intervention.

Changes in Activities of Daily Living (ADL) scores are shown in Figure 5c. At baseline, Patients 5, 7, and 9 were completely dependent on caregivers for daily activities, while Patient 6 exhibited severe dependence. Patients 3 and 8 were also severely dependent, and Patient 4 showed mild dependence. After four weeks of rehabilitation, Patient 2 achieved complete independence in daily living activities. Patients 1 and 4 were mildly dependent; Patients 3, 6, 7, and 8 were moderately dependent; and Patients 5 and 9 remained severely dependent. Overall, the mean ADL score increased from 49.78±39.53 to 66.67±22.50, reflecting improved functional independence.

The changes in National Institutes of Health Stroke Scale (NIHSS) scores are presented in Figure 5d. At baseline, Patients 4 and 9 exhibited moderate neurological impairment. After four weeks of rehabilitation training, both patients improved to mild impairment levels. Overall, the mean NIHSS score decreased from 5.67±4.36 to 3.22±2.99, indicating a reduction in neurological deficit.

Among the four clinical assessment scales, the NIHSS is a negative-direction scale, in which lower scores indicate better outcomes, whereas the Brunnstrom, FMA, and ADL scales are positive-direction scales, in which higher scores reflect improved function. Across all 9 participants, improvements were observed in all clinical indicators to varying degrees. Due to the small sample size, the inclusion of patients at different post-stroke stages, and the absence of a control group, the therapeutic efficacy of the system cannot be established from this uncontrolled preliminary study. Nevertheless, the clinical results demonstrate that the proposed system did not adversely affect patient recovery and was associated with stable or improved functional outcomes. From the perspective of standardized clinical assessments, these findings suggest that the proposed rehabilitation system is feasible and usable under the study conditions.

### 4.3. Usability Evaluation

**Figure 6:**
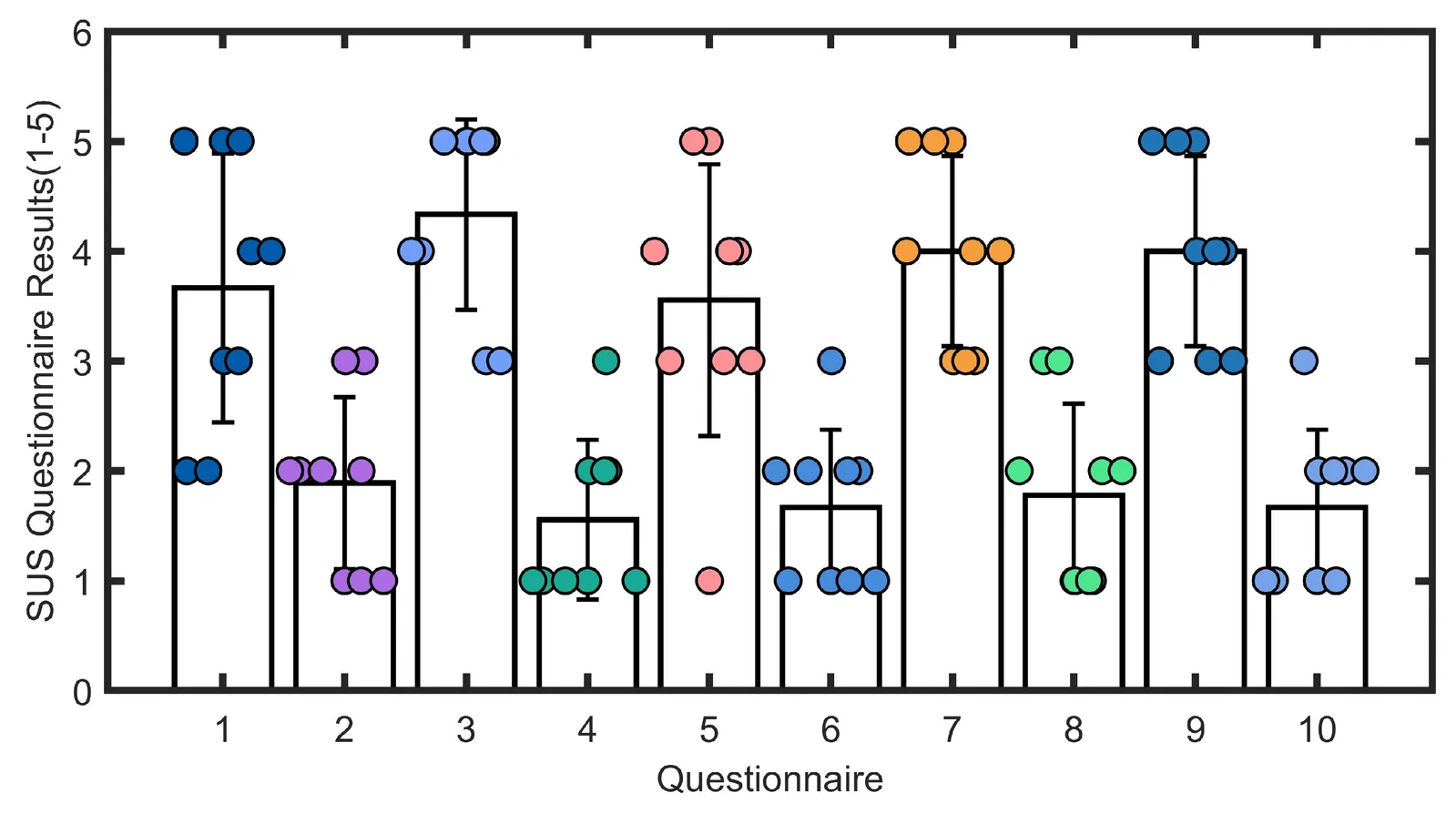
Results of the SUS questionnaire following participant use of the soft hand rehabilitation robot.

The results of the SUS questionnaire are presented in Figure 6. Among the ten SUS items, Questions 1, 3, 5, 7, and 9 are positively worded, whereas Questions 2, 4, 6, 8, and 10 are negatively worded. SUS scoring was performed following standard procedures: for positively worded items, the adjusted score is calculated as (X−1), and for negatively worded items, it is calculated as (5−Y), where *X* and *Y* denote the original Likert-scale responses. The sum of the adjusted scores is multiplied by 2.5 to obtain a total SUS score ranging from 0 to 100, with higher scores indicating better perceived usability. A score of 68 is commonly considered the threshold for acceptable usability.

In this study, the mean score for positively worded items was 3.67±1.22, while the mean score for negatively worded items was 1.02±1.71. The resulting overall SUS score was 74.4, which exceeds the usability threshold. According to the usability rating criteria proposed by Bangor et al. [30], this score corresponds to a usability level classified as “good,” indicating favorable user acceptance of the proposed rehabilitation system.

In response to the open-ended questions regarding system usability, participants most frequently highlighted the soft texture of the gloves and the overall wearing comfort, which motivated continued use of the system. Suggestions for improvement primarily focused on optimizing the external appearance of the soft gloves to enhance their aesthetic appeal and perceived technological sophistication. Furthermore, the majority of participants expressed willingness to continue using the system and indicated acceptance of paying a modest fee for rehabilitation treatment.

### 4.4. Wearability and Durability

During the first week of intervention, the initial three rehabilitation sessions were conducted under direct therapist assistance to help participants don the soft gloves. Subsequently, participants were encouraged to wear the gloves independently. During the rehabilitation period, gloves were worn either independently or with assistance from family members. At the beginning of the intervention, only two participants were able to don the gloves independently; however, after continued training, seven participants achieved independent glove donning. This progression further supports the positive usability outcomes indicated by the SUS results.

Long-term follow-up data revealed that the average donning times for soft gloves fabricated from TPU70D, TPU210D, and TPU420D materials were 16.47±3.97 s, 22.93±3.89 s, and 24.17±3.16 s, respectively. Significant differences in durability were observed among gloves fabricated from different materials. The minimum failure time of gloves fabricated from TPU70D was 7 h (corresponding to seven usage sessions), whereas gloves fabricated from TPU210D exhibited a minimum failure time of 24.3 h. In contrast, gloves fabricated from TPU420D were used for more than 30 h during the experimental period without any observed failure.

These results indicate that the tested higher-stiffness TPU configuration was associated with greater structural stability and durability. However, thicker TPU materials also result in increased initial output force and bending angle of the LSPA, which may adversely affect glove wearability. Taking these trade-offs into account, the soft glove fabricated from TPU420D was selected for use in subsequent studies, as it provides a favorable balance between durability, stability, and functional performance.

## 5. Discussion

This study investigated the feasibility, usability, and effects of a soft hand rehabilitation robot incorporating multimodal feedback through material characterization, system-level evaluation, and a clinical trial. The results demonstrate that the proposed system is feasible and usable for robot-assisted hand rehabilitation in stroke survivors, while also revealing important design trade-offs related to actuator materials, wearability, and durability.

Improvements were observed across all clinical assessment scales, including the Brunnstrom stage, FMA-UE, NIHSS, and ADL. This result is consistent with the previous research findings on rehabilitation treatment [10,31,32]. Although the study was limited by a small sample size and the absence of a control group, the consistent positive trends across these heterogeneous clinical indicators suggest that the proposed robot-assisted intervention did not hinder recovery and may contribute to functional improvement. Notably, several participants exhibited FMA score increases exceeding the minimum clinically important difference (MCID), indicating clinically meaningful gains in upper limb motor function. Improvements in FMA scores were observed as early as the first week of intervention, suggesting that repetitive robot-assisted motion combined with multimodal feedback may facilitate early motor engagement and relearning. Reductions in NIHSS scores indicate decreased neurological impairment, while increased ADL scores reflect enhanced independence in daily living activities. These findings indicate favorable changes across impairment- and function-related measures, although the study design does not permit attribution of these changes to the intervention alone. Nevertheless, due to participant heterogeneity in post-stroke stage and the lack of a comparative control condition, definitive conclusions regarding therapeutic efficacy cannot be drawn. Future randomized controlled trials with larger cohorts are required to quantitatively assess clinical effectiveness.

The SUS results yielded an average score of 74.4, exceeding the commonly accepted usability threshold of 68 and corresponding to a “good” usability rating. This outcome aligns with qualitative feedback from participants, who emphasized the comfort, softness, and ease of use of the soft gloves. The increasing number of participants able to independently don the gloves over time further supports the system’s learnability and practical suitability for both clinical and home-based rehabilitation scenarios. The integration of multimodal feedback—including visual, auditory, force-related, and mirror visual illusion feedback—provided multiple channels for task interaction and may have contributed to sustained user engagement. Interactive rehabilitation tasks, performance-based scoring, and encouraging auditory cues appeared to enhance patient motivation, which is a critical factor in long-term rehabilitation adherence.

Material testing and long-term usage experiments revealed clear trade-offs between compliance, wearability, and durability. Softer TPU materials (e.g., TPU70D) provided greater compliance and shorter donning times but exhibited limited durability and early failure. In contrast, TPU420D demonstrated superior mechanical stability and extended operational lifespan, albeit with increased initial actuation force and stiffness. These results underscore the importance of differentiated material selection in layered soft actuators for rehabilitation applications. Based on the experimental findings, TPU420D was selected for subsequent system iterations, as it offers a favorable balance between durability, stability, and acceptable wearability under therapist supervision.

This study has several limitations. The small sample size and lack of a control group limit the generalizability of the clinical findings. Additionally, the short intervention duration prevents evaluation of long-term rehabilitation effects. Future work will focus on conducting controlled clinical trials with larger populations, optimizing actuator structure to reduce initial actuation force, and incorporating adaptive control strategies tailored to individual patient capabilities.

### Limitations and Future Work

Several limitations should be considered when interpreting these findings. First, the small sample of nine participants limits the generalizability of the usability observations and clinical outcomes. Second, the absence of a control group prevents attribution of the clinical changes to the intervention. Participants differed in baseline impairment and time since stroke, and spontaneous recovery in those enrolled during the acute or early subacute phase may have contributed to changes in clinical scores. Third, robotic assistance and all feedback modalities were delivered together, so the independent or additional benefit of multimodal feedback cannot be established. The qualitative feedback was also not systematically collected by participant and feedback channel. Fourth, the four-week intervention and lack of post-intervention follow-up prevent assessment of whether the observed changes persisted.

The material-use observations lacked standardized failure criteria and matched exposure, and therefore do not establish the comparative durability or optimality of TPU420D. Although the actuator structure and dimensions were retained from our previous work, configuration-specific bending-angle, response-time, and cyclic-fatigue measurements remain necessary. Quantitative sensor and control-system validation was also limited; the programmed pressure ceiling and software stop function should not be interpreted as validated fail-safe mechanisms.

Future work will include larger controlled studies, standardized mechanical testing, and further engineering validation. Robot-only, single-modality, and selected-modality comparisons, together with participant-level feedback, will help assess the contribution of individual feedback channels. Longer follow-up and quantitative measures of task performance, engagement, hand kinematics, and training adherence will also be incorporated.

## 6. Conclusions

This paper presents a soft hand rehabilitation robot system based on multimodal feedback and lattice-structured soft pneumatic actuators (LSPAs). Material characterization, system-level evaluation, and a preliminary study involving nine stroke survivors demonstrated the feasibility of delivering supervised hand rehabilitation training with the system. Group-level clinical scores changed in a favorable direction during the four-week intervention, and the usability assessment indicated generally positive user acceptance and wearing comfort.

Material testing and usage observations suggested a trade-off between compliance and durability and informed the selection of the TPU420D configuration for further evaluation. However, the small sample, absence of a control group, and variation in time since stroke prevent the observed clinical changes from being attributed to the intervention. The present data also cannot establish the independent benefit of multimodal feedback or the comparative superiority of TPU420D. Future work will include standardized actuator testing and larger randomized controlled trials to evaluate clinical efficacy. This study lays the groundwork for future development of soft, wearable, and engaging rehabilitation robots suitable for both clinical and home-based therapy.

## Figures and Tables

**Table 1:** Demographic Information of Patients.

Subject	Gender	Age	Time Post-Stroke	ARAT(66)
S1	M	55	1032 days	53
S2	M	66	3 days	53
S3	M	57	106 days	0
S4	F	33	17 days	0
S5	M	55	22 days	0
S6	F	73	65 days	0
S7	M	52	13 days	0
S8	F	68	90 days	0
S9	F	64	35 days	0

ARAT: Action Research Arm Test.

## Data Availability

The data supporting the findings of this study are included in the article. Further inquiries may be directed to the corresponding author.
